# 1-(4-Methyl­phenyl­sulfon­yl)-5,6-di­nitro-1*H*-indazole

**DOI:** 10.1107/S1600536813034326

**Published:** 2013-12-24

**Authors:** Bassou Oulemda, El Mostapha Rakib, Najat Abbassi, Mohamed Saadi, Lahcen El Ammari

**Affiliations:** a Laboratoire de Chimie Organique et Analytique, Université Sultan Moulay Slimane, Faculté des Sciences et Techniques, Béni-Mellal, BP 523, Morocco; b Laboratoire de Chimie du Solide Appliquée, Faculté des Sciences, Université Mohammed V-Agdal, Avenue Ibn Battouta, BP 1014, Rabat, Morocco

## Abstract

In the title compound, C_14_H_10_N_4_O_6_S, the indazole ring system is almost perpendicular to the tosyl ring, as indicated by the dihedral angle of 89.40 (9)° between their planes. The dihedral angles between the indazole system and the nitro groups are 57.0 (3) and 31.9 (3)°. In the crystal, mol­ecules are linked by C—H⋯O inter­actions, forming chains running along [100].

## Related literature   

For the biological activity of sulfonamides, see: Schmidt *et al.* (2008 ▶); Liu *et al.* (2004 ▶); Ali *et al.* (2008 ▶); Patel *et al.* (1999 ▶); Mosti *et al.* (2000 ▶); Bouissane *et al.* (2006 ▶); Abbassi *et al.* (2012 ▶). For the structures of similar compounds, see: Abbassi *et al.* (2013 ▶); Chicha *et al.* (2013 ▶).
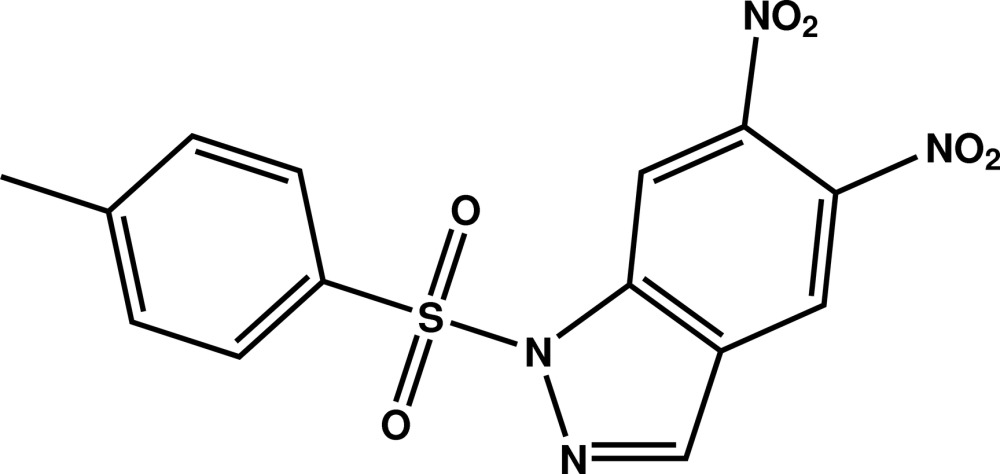


## Experimental   

### 

#### Crystal data  

C_14_H_10_N_4_O_6_S
*M* *_r_* = 362.32
Triclinic, 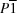

*a* = 7.4125 (3) Å
*b* = 8.5371 (3) Å
*c* = 13.0825 (5) Å
α = 90.401 (2)°
β = 95.707 (2)°
γ = 111.302 (2)°
*V* = 766.66 (5) Å^3^
*Z* = 2
Mo *K*α radiation
μ = 0.25 mm^−1^
*T* = 296 K
0.42 × 0.35 × 0.28 mm

#### Data collection  

Bruker X8 APEX diffractometer
Absorption correction: multi-scan (*SADABS*; Bruker, 2009 ▶) *T*_min_ = 0.693, *T*_max_ = 0.747
19101 measured reflections
3383 independent reflections
2984 reflections with *I* > 2σ(*I*)
*R*_int_ = 0.031

#### Refinement  

*R*[*F* ^2^ > 2σ(*F* ^2^)] = 0.042
*wR*(*F* ^2^) = 0.125
*S* = 1.07
3383 reflections
227 parameters
H-atom parameters constrained
Δρ_max_ = 0.46 e Å^−3^
Δρ_min_ = −0.32 e Å^−3^

### 

Data collection: *APEX2* (Bruker, 2009 ▶); cell refinement: *SAINT* (Bruker, 2009 ▶); data reduction: *SAINT*; program(s) used to solve structure: *SHELXS97* (Sheldrick, 2008 ▶); program(s) used to refine structure: *SHELXL97* (Sheldrick, 2008 ▶); molecular graphics: *ORTEP-3 for Windows* (Farrugia, 2012 ▶); software used to prepare material for publication: *PLATON* (Spek, 2009 ▶) and *publCIF* (Westrip, 2010 ▶).

## Supplementary Material

Crystal structure: contains datablock(s) I. DOI: 10.1107/S1600536813034326/bt6953sup1.cif

Structure factors: contains datablock(s) I. DOI: 10.1107/S1600536813034326/bt6953Isup2.hkl

Supporting information file. DOI: 10.1107/S1600536813034326/bt6953Isup3.cml

Additional supporting information:  crystallographic information; 3D view; checkCIF report

## Figures and Tables

**Table 1:** Hydrogen-bond geometry (Å, °)

*D*—H⋯*A*	*D*—H	H⋯*A*	*D*⋯*A*	*D*—H⋯*A*
C5—H5⋯O5^i^	0.93	2.61	3.175 (2)	120

Symmetry code: (i) 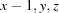.

**Fig. 1.**
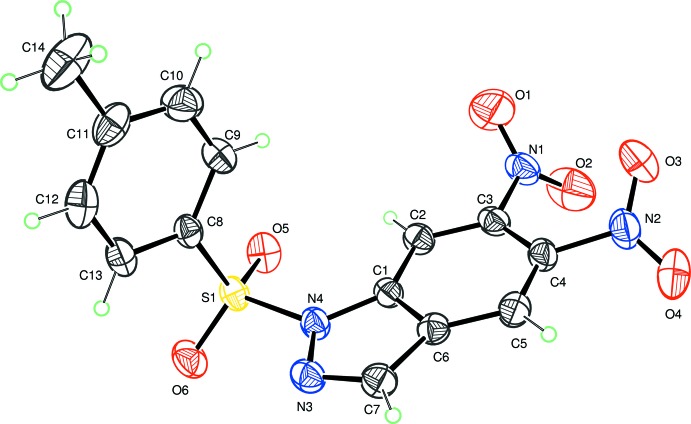
Plot of the molecule of the title compound with the atom-labelling scheme. Displacement ellipsoids are drawn at the 50% probability level. H atoms are represented as small circles.

**Fig. 2.**
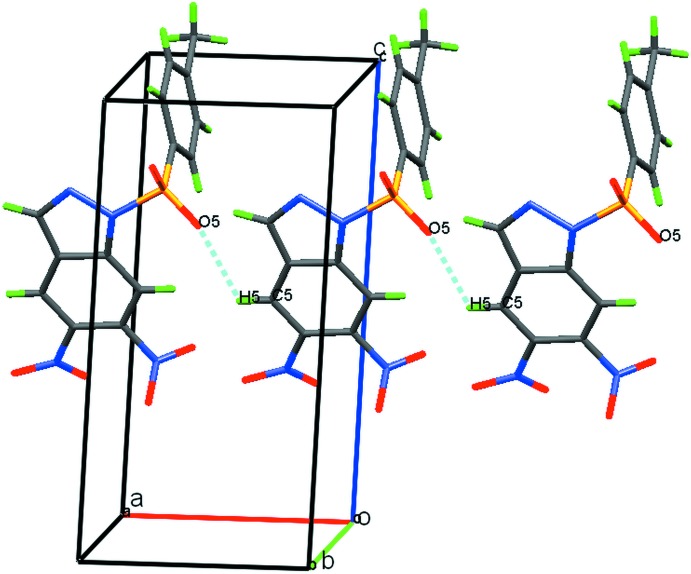
: Partial crystal packing for the title compound showing hydrogen bonds as dashed lines.

## supplementary crystallographic information

### 1. Comment

Indazole derivatives are a versatile class of compounds that have found use in biology, catalysis, and medicinal chemistry (Schmidt *et al.*, 2008). Although rare in nature (Liu *et al.*, 2004; Ali *et al.*, 2008), indazoles exhibit a variety of biological activities such as HIV protease inhibition (Patel *et al.*, 1999), antiarrhythmic and analgesic activities (Mosti *et al.*, 2000), antitumor activity) and antihypertensive properties (Bouissane *et al.*, 2006; Abbassi *et al.*, 2012). The present work is a continuation of the investigation of the sulfonamide derivatives published recently by our team (Abbassi *et al.*, 2013; Chicha *et al.*, 2013).

The molecule of the title compound is built up from an indazole ring system linked to a tosyl ring and to two nitro groups as shown in Fig. 1. The indazole ring system makes dihedral angles of 57.0 (3)° and 31.9 (3)°, with the two plans through the atoms forming the first (N1, O1, O2) and the second (N2, O3, O4) nitro groups, respectively. The plane through the tosyl ring is practically perpendicular to the indazole ring system ring, as indicated by the dihedral angle of 89.40 (9) °. In the crystal, the molecules are linked by a C–H···O interaction to form a one-dimensional chain running along the [100] direction as shown in Fig. 2 and Table 2.

### 2. Experimental

To a stirred solution of 5,6-dinitroindazole (0.5 g, 2.4 mmol) in pyridine (25 ml) was added crude *p*-methylbenzenesulfonyl chloride (0.45 g, 2.4 mmol) over 10 min. The reaction mixture was allowed to attain room temperature and was stirred for further 24 h. The mixture was evaporated under reduced pressure and the residue was purified by silica gel flash column chromatography eluting with dichloromethane. The title compound was recrystallized from acetone. Yield: 56%, m.p.: 307–309 K.

### 3. Refinement

H atoms were located in a difference map and treated as riding with C–H = 0.96 Å and C–H = 0.93 Å for methyl and aromatic, respectively and with *U*_iso_(H) = 1.5 *U*_eq_ for methyl and *U*_iso_(H) = 1.2 *U*_eq_ for aromatic H atoms.

### Crystal data

**Table:**

C_14_H_10_N_4_O_6_S	*Z* = 2
*M**_r_* = 362.32	*F*(000) = 372
Triclinic, *P*1	*D*_x_ = 1.570 Mg m^−^^3^
Hall symbol: -P 1	Mo *K*α radiation, λ = 0.71073 Å
*a* = 7.4125 (3) Å	Cell parameters from 3383 reflections
*b* = 8.5371 (3) Å	θ = 2.6–27.1°
*c* = 13.0825 (5) Å	µ = 0.25 mm^−^^1^
α = 90.401 (2)°	*T* = 296 K
β = 95.707 (2)°	Block, colourless
γ = 111.302 (2)°	0.42 × 0.35 × 0.28 mm
*V* = 766.66 (5) Å^3^	

### Data collection

**Table:**

Bruker X8 APEX diffractometer	3383 independent reflections
Radiation source: fine-focus sealed tube	2984 reflections with *I* > 2σ(*I*)
Graphite monochromator	*R*_int_ = 0.031
φ and ω scans	θ_max_ = 27.1°, θ_min_ = 2.6°
Absorption correction: multi-scan (*SADABS*; Bruker, 2009)	*h* = −9→9
*T*_min_ = 0.693, *T*_max_ = 0.747	*k* = −10→10
19101 measured reflections	*l* = −16→16

### Refinement

**Table:**

Refinement on *F*^2^	Secondary atom site location: difference Fourier map
Least-squares matrix: full	Hydrogen site location: inferred from neighbouring sites
*R*[*F*^2^ > 2σ(*F*^2^)] = 0.042	H-atom parameters constrained
*wR*(*F*^2^) = 0.125	*w* = 1/[σ^2^(*F*_o_^2^) + (0.0641*P*)^2^ + 0.3549*P*] where *P* = (*F*_o_^2^ + 2*F*_c_^2^)/3
*S* = 1.07	(Δ/σ)_max_ < 0.001
3383 reflections	Δρ_max_ = 0.46 e Å^−^^3^
227 parameters	Δρ_min_ = −0.32 e Å^−^^3^
0 restraints	Extinction correction: *SHELXL97* (Sheldrick, 2008), Fc^*^=kFc[1+0.001xFc^2^λ^3^/sin(2θ)]^-1/4^
Primary atom site location: structure-invariant direct methods	Extinction coefficient: 0.023 (4)

### Special details

**Table:**

Geometry. All e.s.d.'s (except the e.s.d. in the dihedral angle between two l.s. planes) are estimated using the full covariance matrix. The cell e.s.d.'s are taken into account individually in the estimation of e.s.d.'s in distances, angles and torsion angles; correlations between e.s.d.'s in cell parameters are only used when they are defined by crystal symmetry. An approximate (isotropic) treatment of cell e.s.d.'s is used for estimating e.s.d.'s involving l.s. planes.
Refinement. Refinement of *F*^2^ against all reflections. The weighted *R*-factor *wR* and goodness of fit *S* are based on *F*^2^, conventional *R*-factors *R* are based on *F*, with *F* set to zero for negative *F*^2^. The threshold expression of *F*^2^ > σ(*F*^2^) is used only for calculating *R*-factors(gt) *etc*. and is not relevant to the choice of reflections for refinement. *R*-factors based on *F*^2^ are statistically about twice as large as those based on *F*, and *R*- factors based on all data will be even larger.

### Fractional atomic coordinates and isotropic or equivalent isotropic displacement parameters (Å^2^)

**Table:**

	*x*	*y*	*z*	*U*_iso_*/*U*_eq_	
C1	0.0249 (2)	0.1414 (2)	0.35023 (12)	0.0319 (3)	
C2	0.1588 (3)	0.2338 (2)	0.43202 (13)	0.0365 (4)	
H2	0.2924	0.2632	0.4312	0.044*	
C3	0.0814 (3)	0.2782 (2)	0.51328 (13)	0.0370 (4)	
C4	−0.1209 (3)	0.2326 (2)	0.51564 (13)	0.0389 (4)	
C5	−0.2510 (3)	0.1422 (2)	0.43547 (14)	0.0395 (4)	
H5	−0.3845	0.1121	0.4373	0.047*	
C6	−0.1768 (2)	0.0965 (2)	0.35082 (13)	0.0340 (4)	
C7	−0.2599 (3)	0.0120 (2)	0.25344 (14)	0.0401 (4)	
H7	−0.3929	−0.0317	0.2322	0.048*	
C8	0.2592 (2)	0.2835 (2)	0.12063 (13)	0.0341 (4)	
C9	0.3100 (3)	0.4449 (2)	0.16444 (15)	0.0459 (4)	
H9	0.3465	0.4669	0.2347	0.055*	
C10	0.3047 (3)	0.5715 (3)	0.10045 (19)	0.0540 (5)	
H10	0.3356	0.6796	0.1286	0.065*	
C11	0.2545 (3)	0.5414 (3)	−0.00455 (17)	0.0494 (5)	
C12	0.2060 (3)	0.3792 (3)	−0.04551 (16)	0.0523 (5)	
H12	0.1725	0.3578	−0.1160	0.063*	
C13	0.2064 (3)	0.2490 (3)	0.01622 (14)	0.0434 (4)	
H13	0.1718	0.1404	−0.0118	0.052*	
C14	0.2506 (4)	0.6822 (4)	−0.0728 (2)	0.0757 (8)	
H14A	0.3662	0.7798	−0.0550	0.114*	
H14B	0.1380	0.7089	−0.0635	0.114*	
H14C	0.2451	0.6474	−0.1434	0.114*	
N1	0.2229 (3)	0.3722 (2)	0.59984 (13)	0.0499 (4)	
N2	−0.1975 (3)	0.2944 (2)	0.60016 (14)	0.0537 (5)	
N3	−0.1278 (2)	0.00328 (19)	0.19798 (11)	0.0396 (3)	
N4	0.0489 (2)	0.07838 (19)	0.25754 (11)	0.0361 (3)	
O1	0.3506 (3)	0.5014 (3)	0.58078 (16)	0.0959 (8)	
O2	0.2091 (4)	0.3122 (3)	0.68252 (13)	0.0874 (7)	
O3	−0.0949 (3)	0.4309 (2)	0.64245 (15)	0.0780 (6)	
O4	−0.3607 (3)	0.2146 (3)	0.61839 (17)	0.0944 (7)	
O5	0.4057 (2)	0.1788 (2)	0.28403 (11)	0.0491 (4)	
O6	0.2305 (2)	−0.02753 (18)	0.14045 (12)	0.0538 (4)	
S1	0.25624 (6)	0.11985 (6)	0.20019 (3)	0.03714 (16)	

### Atomic displacement parameters (Å^2^)

**Table:**

	*U*^11^	*U*^22^	*U*^33^	*U*^12^	*U*^13^	*U*^23^
C1	0.0385 (8)	0.0306 (8)	0.0282 (8)	0.0145 (7)	0.0030 (6)	0.0020 (6)
C2	0.0380 (9)	0.0381 (9)	0.0325 (8)	0.0140 (7)	−0.0008 (7)	−0.0007 (7)
C3	0.0487 (10)	0.0311 (8)	0.0301 (8)	0.0145 (7)	0.0004 (7)	0.0002 (6)
C4	0.0540 (11)	0.0348 (9)	0.0329 (9)	0.0200 (8)	0.0128 (8)	0.0052 (7)
C5	0.0400 (9)	0.0410 (9)	0.0396 (9)	0.0160 (8)	0.0096 (7)	0.0059 (7)
C6	0.0357 (8)	0.0326 (8)	0.0337 (8)	0.0126 (7)	0.0028 (7)	0.0038 (6)
C7	0.0359 (9)	0.0423 (9)	0.0384 (9)	0.0112 (7)	−0.0012 (7)	−0.0009 (7)
C8	0.0316 (8)	0.0409 (9)	0.0322 (8)	0.0157 (7)	0.0053 (6)	−0.0024 (7)
C9	0.0515 (11)	0.0454 (10)	0.0396 (10)	0.0172 (9)	0.0019 (8)	−0.0073 (8)
C10	0.0575 (13)	0.0419 (11)	0.0643 (14)	0.0192 (9)	0.0102 (11)	0.0015 (10)
C11	0.0371 (10)	0.0604 (12)	0.0581 (12)	0.0230 (9)	0.0183 (9)	0.0208 (10)
C12	0.0515 (11)	0.0751 (14)	0.0351 (10)	0.0277 (11)	0.0092 (8)	0.0092 (9)
C13	0.0475 (10)	0.0515 (11)	0.0322 (9)	0.0193 (9)	0.0057 (8)	−0.0061 (8)
C14	0.0625 (15)	0.0889 (19)	0.094 (2)	0.0421 (14)	0.0337 (14)	0.0504 (16)
N1	0.0650 (11)	0.0446 (9)	0.0355 (9)	0.0168 (8)	−0.0028 (8)	−0.0076 (7)
N2	0.0717 (12)	0.0547 (10)	0.0418 (9)	0.0282 (9)	0.0205 (9)	0.0028 (8)
N3	0.0415 (8)	0.0407 (8)	0.0332 (8)	0.0129 (7)	−0.0027 (6)	−0.0040 (6)
N4	0.0360 (7)	0.0409 (8)	0.0298 (7)	0.0127 (6)	0.0015 (6)	−0.0040 (6)
O1	0.0898 (15)	0.0859 (14)	0.0680 (12)	−0.0171 (12)	−0.0005 (11)	−0.0216 (11)
O2	0.1383 (19)	0.0776 (12)	0.0387 (9)	0.0375 (13)	−0.0184 (10)	0.0019 (8)
O3	0.1032 (15)	0.0646 (11)	0.0696 (11)	0.0315 (10)	0.0245 (11)	−0.0176 (9)
O4	0.0930 (15)	0.0952 (15)	0.0867 (14)	0.0130 (12)	0.0569 (13)	−0.0114 (12)
O5	0.0403 (7)	0.0691 (9)	0.0438 (7)	0.0289 (7)	−0.0017 (6)	0.0018 (7)
O6	0.0701 (10)	0.0486 (8)	0.0543 (9)	0.0339 (7)	0.0137 (7)	−0.0048 (7)
S1	0.0394 (3)	0.0435 (3)	0.0349 (2)	0.0227 (2)	0.00423 (18)	−0.00191 (18)

### Geometric parameters (Å, º)

**Table:**

C1—N4	1.377 (2)	C10—C11	1.384 (3)
C1—C2	1.400 (2)	C10—H10	0.9300
C1—C6	1.403 (2)	C11—C12	1.387 (3)
C2—C3	1.370 (2)	C11—C14	1.509 (3)
C2—H2	0.9300	C12—C13	1.379 (3)
C3—C4	1.409 (3)	C12—H12	0.9300
C3—N1	1.472 (2)	C13—H13	0.9300
C4—C5	1.368 (3)	C14—H14A	0.9600
C4—N2	1.469 (2)	C14—H14B	0.9600
C5—C6	1.397 (2)	C14—H14C	0.9600
C5—H5	0.9300	N1—O2	1.198 (2)
C6—C7	1.425 (2)	N1—O1	1.211 (3)
C7—N3	1.298 (2)	N2—O4	1.202 (3)
C7—H7	0.9300	N2—O3	1.227 (3)
C8—C13	1.382 (2)	N3—N4	1.383 (2)
C8—C9	1.392 (3)	N4—S1	1.6992 (15)
C8—S1	1.7429 (18)	O5—S1	1.4245 (14)
C9—C10	1.381 (3)	O6—S1	1.4186 (14)
C9—H9	0.9300		
			
N4—C1—C2	132.03 (16)	C10—C11—C14	120.6 (2)
N4—C1—C6	105.65 (15)	C12—C11—C14	120.8 (2)
C2—C1—C6	122.32 (16)	C13—C12—C11	121.32 (19)
C3—C2—C1	116.04 (16)	C13—C12—H12	119.3
C3—C2—H2	122.0	C11—C12—H12	119.3
C1—C2—H2	122.0	C12—C13—C8	118.61 (19)
C2—C3—C4	122.37 (16)	C12—C13—H13	120.7
C2—C3—N1	115.68 (17)	C8—C13—H13	120.7
C4—C3—N1	121.92 (16)	C11—C14—H14A	109.5
C5—C4—C3	121.29 (16)	C11—C14—H14B	109.5
C5—C4—N2	117.78 (18)	H14A—C14—H14B	109.5
C3—C4—N2	120.69 (17)	C11—C14—H14C	109.5
C4—C5—C6	117.80 (17)	H14A—C14—H14C	109.5
C4—C5—H5	121.1	H14B—C14—H14C	109.5
C6—C5—H5	121.1	O2—N1—O1	124.8 (2)
C5—C6—C1	120.16 (16)	O2—N1—C3	118.03 (18)
C5—C6—C7	134.80 (17)	O1—N1—C3	117.08 (18)
C1—C6—C7	105.00 (15)	O4—N2—O3	124.3 (2)
N3—C7—C6	111.90 (16)	O4—N2—C4	118.2 (2)
N3—C7—H7	124.1	O3—N2—C4	117.26 (19)
C6—C7—H7	124.1	C7—N3—N4	106.16 (14)
C13—C8—C9	121.71 (18)	C1—N4—N3	111.21 (14)
C13—C8—S1	119.26 (14)	C1—N4—S1	128.92 (12)
C9—C8—S1	119.01 (14)	N3—N4—S1	118.15 (11)
C10—C9—C8	118.03 (18)	O6—S1—O5	121.02 (9)
C10—C9—H9	121.0	O6—S1—N4	106.26 (8)
C8—C9—H9	121.0	O5—S1—N4	103.11 (8)
C9—C10—C11	121.7 (2)	O6—S1—C8	110.38 (9)
C9—C10—H10	119.2	O5—S1—C8	110.65 (9)
C11—C10—H10	119.2	N4—S1—C8	103.62 (8)
C10—C11—C12	118.67 (19)		

### Hydrogen-bond geometry (Å, º)

**Table:**

*D*—H···*A*	*D*—H	H···*A*	*D*···*A*	*D*—H···*A*
C5—H5···O5^i^	0.93	2.61	3.175 (2)	120

Symmetry code: (i) *x*−1, *y*, *z*.
